# Selecting RAD-Seq Data Analysis Parameters for Population Genetics: The More the Better?

**DOI:** 10.3389/fgene.2019.00533

**Published:** 2019-05-29

**Authors:** Natalia Díaz-Arce, Naiara Rodríguez-Ezpeleta

**Affiliations:** Marine Research Division, AZTI, Sukarrieta, Spain

**Keywords:** restriction site-associated DNA sequencing, PCR clones, stacks parameters, SNP filtering, *de novo* assembly

## Abstract

Restriction site-associated DNA sequencing (RAD-seq) has become a powerful and widely used tool in molecular ecology studies as it allows to cost-effectively recover thousands of polymorphic sites across individuals of non-model organisms. However, its successful implementation in population genetics relies on correct data processing that would minimize potential loci-assembly biases and consequent genotyping error rates. RAD-seq data processing when no reference genome is available involves the assembly of hundreds of thousands high-throughput sequencing reads into orthologous loci, for which various key parameter values need to be selected by the researcher. Previous studies exploring the effect of these parameter values found or assumed that a larger number of recovered polymorphic loci is associated with a better assembly. Here, using three RAD-seq datasets from different species, we explore the effect of read filtering, loci assembly and polymorphic site selection on number of markers obtained and genetic differentiation inferred using the Stacks software. We find (i) that recovery of higher numbers of polymorphic loci is not necessarily associated with higher genetic differentiation, (ii) that the presence of PCR duplicates, selected loci assembly parameters and selected SNP filtering parameters affect the number of recovered polymorphic loci and degree of genetic differentiation, and (iii) that this effect is different in each dataset, meaning that defining a systematic universal protocol for RAD-seq data analysis may lead to missing relevant information about population differentiation.

## Introduction

Restriction site-associated DNA sequencing (RAD-seq) (Baird et al., 2008) and related methods (Davey et al., 2011) are revolutionizing the fields of ecological and evolutionary genomics (Davey and Blaxter, 2010; Andrews et al., 2016). These approaches consist in subsampling putative homologous regions from the genome of several individuals with the aim of discovering and genotyping thousands of variable genetic markers that can be used for evolutionary, phylogenomic and population structure studies among others (Andrews et al., 2016). RAD-seq is particularly relevant for studies focused on species for which no genomic resources are available as it allows to cost-effectively discover thousands of genome-wide SNPs while genotyping them in hundreds of individuals performing *de novo* alignment of the reads (Davey et al., 2011). Thus, the number of studies relying on RAD-seq or related approaches for assessing population differentiation is increasing exponentially (Davey and Blaxter, 2010; Andrews et al., 2016).

As for other approaches relying on high-throughput sequencing, data processing is one of the major challenges of reduced representation sequencing studies. The hundreds of thousands short reads need to be assembled into putative alleles and then into putative orthologous loci, for which some assumptions need to be made (Catchen et al., 2013; Davey et al., 2013; Eaton, 2014; Sovic et al., 2015). Several software packages for assembling orthologous loci and typing variant positions from reduced representation sequencing data have been developed [i.e., PyRAD (Eaton, 2014), AftrRAD (Sovic et al., 2015), Rainbow (Wu et al., 2012), RADtools (Baxter et al., 2011), RADProc (Nadukkalam Ravindran et al., 2019), and Stacks (Catchen et al., 2013)]. Among them, Stacks is one of the most widely used programs and for which procedures for several applications have been established (Rochette and Catchen, 2017). The program comprises several modules for read preprocessing (*process_radtags*), read merging into loci within individuals (*ustacks* for *de novo* merging and *pstacks* for reference-based merging), merging loci between individuals (*cstacks*) and loci and variant selection for further analysis (*genotypes* and *populations*). Read merging into loci within individuals relies on two main parameters: the minimum required read coverage depth to form a stack or group of identical reads (*m*), the maximum number of mismatches allowed between stacks or groups of identical reads to be considered as different alleles of the same locus (*M*). Loci merging between individuals relies on one main parameter: the maximum number of mismatches between loci from different individuals to be considered homologs (*n*). Additional pipelines are available to complement Stacks data processing steps, such as *clone_filter*, for filtering PCR clones, that is, identical sequence fragments generated during the amplification process required for RAD-seq library generation, when paired-ends are available.

How to properly select the read processing parameters for obtaining a meaningful set of markers from RAD-seq data is a largely discussed issue, and several studies have examined the effect of different parameters on the number of obtained loci (Catchen et al., 2013; Paris et al., 2017), SNP call and genotyping error rate (Mastretta-Yanes et al., 2015; O’Leary et al., 2018), resolution power of derived phylogeny (Cruaud et al., 2014; Harvey et al., 2015; Díaz-Arce et al., 2016) and population genetic and evolutionary inferences (Puebla et al., 2014; Rodríguez-Ezpeleta et al., 2016, 2017; Shafer et al., 2017). From a theoretical point of view and from results obtained by these studies, the anticipated effect of under or over estimating each of the above mentioned Stacks parameters can be inferred: for example, setting too low or too high *m* values might result in an under or an over-merging of reads, respectively (Catchen et al., 2013). There are additional biases inherent to RAD-seq data that have been discussed, such as allele dropout (Arnold et al., 2013; Gautier et al., 2013; O’Leary et al., 2018) and false genotypes due to the presence of PCR clones (Davey et al., 2013; Andrews et al., 2014; Tin et al., 2015; O’Leary et al., 2018). These biases could potentially lead into high genotyping error rates, which could be reduced by a correct data assembly and filtering (Hendricks et al., 2018).

In search of a consensus for parameter selection, two studies applied systematic iterations of the main parameters within Stacks and defined the optimal parameter set as that which minimizes genotyping errors and maximizes number of shared loci (Mastretta-Yanes et al., 2015) or only the latter (Paris et al., 2017). Yet, obtaining the maximum number of shared loci among individuals included in our study is not indicative of the accuracy of orthology assignment or SNP calling, neither of the meaningful genetic information contained in the dataset. Indeed, none of these studies tested the effect of the different parameter combinations on the derived population genetics analyses, which can also be affected by the subsequent SNP filtering steps (Roesti et al., 2012; De la Cruz and Raska, 2014). For example, population structure inferences based on SNPs filtered by different minimum allele frequency (MAF) threshold values by De la Cruz and Raska (2014) derived into different patterns of differentiation.

Here we have, used data from three published studies to explore the effect of removing PCR clones and of using alternative values of the main Stacks parameters and of MAF thresholds for SNP selection on the number of obtained shared markers and on population genetic inferences. The aim of the study is to analyze the importance of parameter setting during the *de novo* RAD-seq data analysis, and to test the derived effects on population differentiation inferences. Our results show that maximizing the number of obtained shared polymorphic loci in the dataset does not necessarily provide the strongest genetic differentiation signal and suggest that a systematic Stacks parameter selection method might limit population differentiation power of the dataset.

## Materials and Methods

### Datasets

We selected a subset of individuals of European green crab (*Carcinus maenas*), Atlantic mackerel (*Scomber scombrus*), and Atlantic deep-sea scallop (*Placopecten magellanicus*) from three previous studies (Rodríguez-Ezpeleta et al., 2016; Jeffery et al., 2017; Van Wyngaarden et al., 2017) for which RAD-seq data are publicly available (Table 1). Libraries for all three datasets were prepared following the same protocol (Etter et al., 2011) using the *SbfI* restriction enzyme, but with a variable number of PCR cycles for RAD-tag amplification (Table 1). The Atlantic mackerel dataset consists of individuals from four locations of which all pairs show genetic differentiation: larger *F*_ST_ values are observed between Atlantic Ocean and Mediterranean Sea locations. The green crab and scallop datasets include individuals from, respectively, four and five locations along the East coast of North America (latitude 39–49°N). In both species, northern and southern locations (separated at latitude 45°N) are genetically differentiated. No differentiation is found within green crab northern or southern, nor within scallop southern locations. However, genetic differentiation is observed within northern scallop locations.

**Table 1:** For each species, number of individuals analyzed per location and population, number of PCR-cycles used for library building, average number and standard deviation (SD) of forward reads retained per individual and average depth coverage per locus when applying *m* = 3, *M* = 2 parameters, before (above) and after (below) removing PCR clones.

Species	Location	Population	*n*	PCR-cycles	Average number of reads	Average depth coverage (*m* = 3, *M* = 2)	NCBI BioProject
European green crab (*Carcinus maenas*)	Brudenell River	North	22	14	6,750,558 (SD: 2,594,048) 2,389,818 (SD: 767,861)	221x 93.6x	PRJNA377723
	Cole Harbour	North	22	14			
	Campobello Island	South	22	14			
	Tuckerton	South	22	14			
Atlantic mackerel (*Scomber scombrus*)	East Canada	West Atlantic	29	14	3,161,222 (SD: 1,630,037) 1,905,752 (SD: 902,165)	43x 33x	PRJNA310297
	Bay of Biscay	East Atlantic	22	14			
	Adriatic Sea	East Mediterranean	20	14			
	Western Mediterranean	West Mediterranean	16	14			
Deep sea scallop (*Placopecten magellanicus*)	Sunnyside	North	20	13	7,198,343 (SD: 1,807,699) 1,924,472 (SD: 1,433,721)	171x 58.3x	PRJNA340326
	Little Bay	North	21	18			
	Magdalen Islands	North	21	18			
	Gulf of Main	South	20	18			
	Browns Bank	South	22	13			

*Bioproject number for Data Availability of each dataset is included.*

### RAD-Seq Data Preprocessing

Raw reads were processed with Stacks v1.44 (Catchen et al., 2013). Quality filtering and demultiplexing was performed using *process_radtags* truncating all reads to 90 nucleotides to avoid the lower quality bases at the end of the read. PCR clones were removed applying *clone_filter* to reads whose forward and reverse pairs passed quality filtering. Using separately non-clone-filtered data (i.e., all forward reads passing quality filtering, even if their reverse pair failed) and clone-filtered data (i.e., single representatives of each PCR clone), putative orthologous loci (RAD tags) per individual were assembled using *ustacks*. The minimum number of identical cleaned sequence reads used to form a stack (*m*) was set iteratively from 2 to 5, and the maximum number of nucleotide mismatches allowed between stacks before merging two or more stacks into a locus (*M*) set to 2 or 4. Reads not included in primary stacks during individual RAD loci formation (secondary reads) were subsequently incorporated to increase primary stack depth allowing a maximum nucleotide mismatch (*N*) of *M* + 2 (default).

Catalogs of RAD loci were assembled using *cstacks* with a maximum number of nucleotide mismatches allowed between loci while merging them into the catalog (*n*) of 3 (for *M* = 2) or 6 (for *M* = 4). In sum, for each species, 16 catalogs were generated combining the use or not of PCR clones, the use of four different *m* values and the use of two different combinations of *M* and *n* values. Matches of individual RAD loci to the catalog were searched using *sstacks* and SNPs present in RAD loci found in at least 75% of the individuals under study were selected using *populations*. One additional catalog was generated per species following the “r80 rule” (Paris et al., 2017), which consists in selecting the *m*, *M*, and *n* parameter values that provide the maximum number of polymorphic loci present in at least the 80% of the individuals; the process consists in (i) selecting the optimal *m* value (among values ranging from 2 to 7) for *M* = 2, *n* = 0, (ii) selecting the optimal *M* value (among values ranging from 1 to 5) for the *m* value optimized previously and *N* = 0 and iii) selecting the optimal *n* value (among *M* − 1, *M*, and *M* + 1) for the *m* and *M* values optimized previously. Optimum Stacks parameters following the “r80 rule” were *m* = 3, *M* = 4, *n* = 4 for mackerel, *m* = 6, *M* = 1, *n* = 1 for scallop, and *m* = 7, *M* = 2, *n* = 2 for the green crab datasets.

### SNP Genotype Table Generation and Calculations of Population Differentiation

Using PLINK version 1.07 (Purcell et al., 2007), individuals with a genotyping rate smaller than 0.4 where removed, and SNPs with a genotyping rate smaller than 0.99 (for mackerel) and 0.85 (for scallop and green crab) were removed. SNPs were filtered according to a minimum minor allele frequency (MAF) of 0.01, 0.05, or 0.10. The resulting 153 genotype datasets (three per catalog) were generated and exported to GENEPOP (Rousset, 2008) format using PGDSpider version 2.0.8.3 (Lischer and Excoffier, 2011). Overall fixation index (*F*_ST_) per population pair was calculated following the Weir and Cockerham (1984) formulation as implemented in *Genepop 4.3* (Rousset, 2008). In addition, *F*_ST_ was calculated for each catalog and pair using a subset of 2000 SNPs to test the possible effect of the number of SNPs included in the calculation.

## Results and Discussion

### Effect of PCR Clones on RAD-Loci Assembly

**FIGURE 1:**
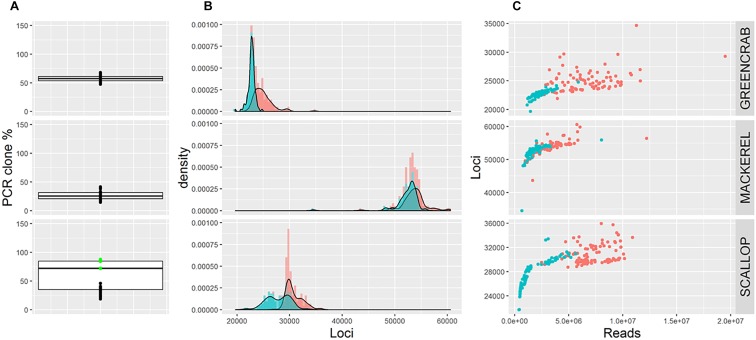
**(A)** Boxplots showing percentage of PCR clones per individual. Green dots represent scallop individuals whose libraries were generated using 18 PCR cycles. **(B)** Frequency distribution of the number of loci per individual before (red) and after (blue) removing PCR clones. **(C)** Number of retained reads and assembled loci per individual before (red) and after (blue) removing PCR clones. Note that **B** and **C** show number of loci estimated using *m* = 5, *M* = 4; alternative parameter combinations produce equivalent results (see Supplementary Figures S2, S3, S4).

Average percentage of PCR clones per species differ (Figure 1A), being 27.1% for mackerel, 57.2% for green crab, and 58.1% for scallop. Whereas in mackerel and green crab the number of PCR clones is similar across individuals, in scallop, groups of samples processed using 13 or 18 PCR cycles can be distinguished (23 and 82% of clone reads, respectively, Figure 1A). Thus, average PCR clone percentages increase with number of PCR cycles, as expected (Andrews et al., 2016). Yet, although both mackerel and green crab datasets were generated using 14 PCR cycles, mackerel shows a lower percentage of clonal reads. The use of different amounts of starting material could have an effect on presence proportions of these PCR clones (Davey et al., 2011; Andrews et al., 2016), but here we reject this hypothesis as green crab libraries were generated from more starting DNA than the mackerel libraries. Instead, this could be explained by the larger number of reads for green crab (Table 1) combined with a lower number of *SbfI* cut sites, inferred from a lowest number of loci (Figure 1B), which makes presence of PCR clones more likely.

The maximum possible number of correct RAD loci per individual depends on the number of cut sites for the restriction enzyme of choice present in the genome of the species under study. Reaching this maximum number depends on the number of reads sequenced, so that a minimum coverage per loci is ensured. Here, although the average number of loci obtained per individual differs per species, in all cases the number of loci increases with sequencing depth until a certain value of convergence (Figure 1C). This convergence suggests that this maximum number is reached for each species. After removing PCR clones the number of loci per individual is less variable and the maximum total number of RAD loci is more clearly identified (Figure 1B,C), suggesting that when PCR clones are included artefactual loci might appear. Indeed, average number of assembled loci per individual is lower when removing PCR clones, a difference that is less pronounced in mackerel (lower average percentage of PCR clones per individual). Interestingly, in scallop, numbers of loci per individual follow the same bimodal distribution observed for percentages of PCR clones, suggesting that the clone percentage affects the number of inferred loci, and that removing clone reads only partially corrects this effect (Figure 1B and Supplementary Figure S1). The PCR clone percentages found in our three examples are in the range of what it is found in other reduced-representation library sequencing datasets (Andrews et al., 2014, 2016), suggesting that the effects we observe can be extrapolated to other studies.

### Effect of RAD-Loci Assembly Parameters and MAF Thresholds on Number of Selected Loci and SNPs

**FIGURE 2:**
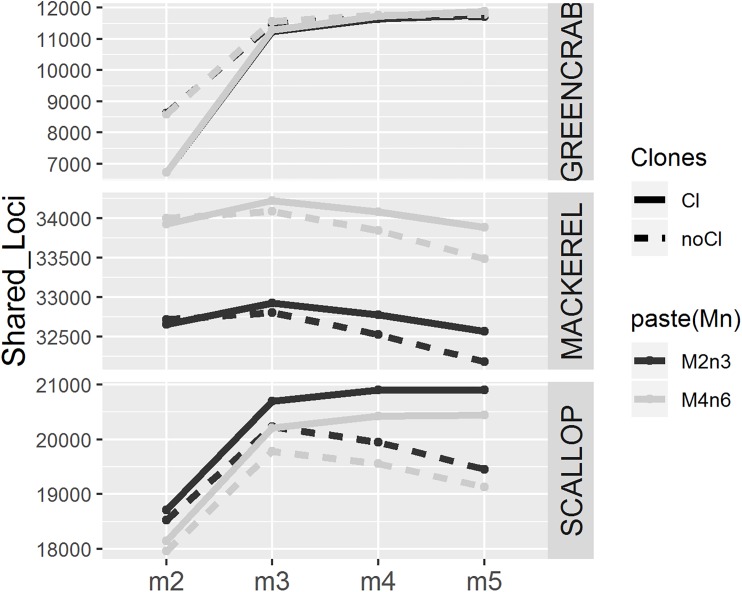
Number of polymorphic loci present in at least 75% of the individuals for different values of *m* (*x* axis), using different combinations of *M* and *n* parameters (*M* = 2, *n* = 3 in black and *M* = 4, *n* = 6 in gray), before (solid line) and after (dotted line) removing PCR clones.

As expected (Paris et al., 2017), increasing values of *m* result in lower and more homogeneous numbers of individual loci recovered across individuals, particularly before filtering PCR clones (Supplementary Figures S2, S3, S4). This is because lower values of *m* result in loci assembled from low coverage haplotypes, which could be generated from PCR or sequencing errors. In all cases, the number of shared loci is higher when increasing *m* from 2 to 3, although this effect is less pronounced in clone filtered catalogs, where PCR derived erroneous reads have been likely removed (Figure 2 and Supplementary Figure S5). As shown, allowing a minimum stack depth parameter of *m* = 2 results in highest number of loci per individual (Supplementary Figures S2, S3, S4), which would increase the chance between individual loci to match. At the same time, this would increase the chance for more than one individual locus to collapse into the same catalog locus and vice versa, consequently, decreasing the number of shared loci. Yet, when increasing *m* from 3 to 4 and 5, the number of shared loci decreases or increases depending on the dataset, and on the removal or not of PCR clones (Figure 2 and Supplementary Figure S5). In their study, Paris et al. (2017) also found that the number of polymorphic loci increased from *m* = 2 to *m* = 3 and decreased when using higher values of *m*. Here, in the mackerel catalogs and the PCR clone filtered scallop catalogs, for which also number of shared polymorphic loci decrease with high values of *m*, show average coverages per locus similar to those included in Paris et al. (2017; Table 1). Therefore, one possible explanation for the decrease in the number of shared loci after peaking at certain value of *m* could be missing loci (being harder for a locus to be shared among individuals) and/or haplotypes (being harder to find orthologous loci with lower number of alleles recovered) with lowest coverages. Interestingly, in the mackerel dataset before removing clones, while the number of polymorphic loci decreases with values of *m* higher than 3 (Figure 2), the total number of shared loci (both monomorphic and polymorphic) still increases (Supplementary Figure S5), which could be explained by skewed haplotype coverages due to the presence of PCR clones, which would lead into heterozygotes to appear as homozygotes (Andrews et al., 2016). None of these two measures (number of shared total or polymorphic loci) alone does necessarily indicate a more realistic assemblage. Besides, in this case, the values of the *m* parameter that provides the highest number of polymorphic loci and the highest number of total shared loci is not the same in all datasets.

Changing *M* and *n* parameters from *M* = 2, *n* = 3 to *M* = 4, *n* = 6 makes the number of shared loci increase and decrease in mackerel and scallop datasets, respectively, while we observed almost no differences in the green crab dataset. In mackerel, it has been shown that while increasing *n* from 3 to 6 would make more RAD loci merge in the same catalog locus reducing the number of common loci found, increasing *M* from 2 to 4 increases the number of shared loci, as common loci would be more easily found with higher number of alleles per locus (Rodríguez-Ezpeleta et al., 2016). The separated effect of *M* and *n* parameters has not been tested in this study and there may be different causes for variation.

**FIGURE 3:**
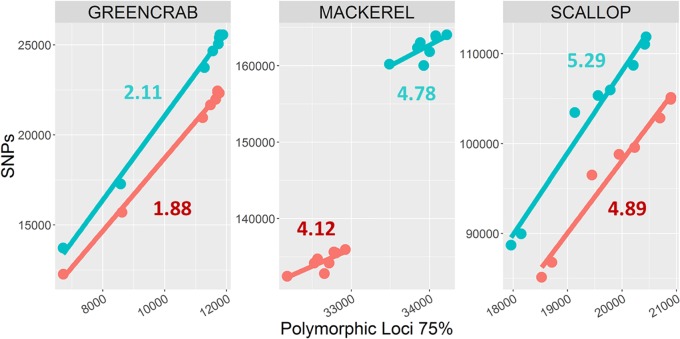
Numbers of shared polymorphic loci and derived SNPs. Dots represent catalogs built using *M* = 2, *n* = 3 (blue), and *M* = 4, *n* = 6 (red) combinations. Each color includes eight dots, corresponding to *m* = 2, *m* = 3, *m* = 4, and *m* = 5, and PCR clone filtered/non-filtered catalogs. Numbers represent average number of SNPs per shared polymorphic loci in *M* = 2, *n* = 3 (blue) and *M* = 4, *n* = 6 (red) catalogs.

The number of shared SNPs in general increased with increasing number of shared polymorphic loci, regardless the different *m* values and the use or exclusion of PCR clones. When increasing the *M* and *n* parameters from *M* = 2, *n* = 3 to *M* = 4, *n* = 6, both the total number of SNPs and average number of SNPs per shared polymorphic locus always increases (Figure 3), including the scallop and green crab catalogs, for which the number of shared polymorphic loci respectively decreases and remains nearly identical. On the other hand, the green crab dataset shows the lowest number of SNPs per locus, followed by the mackerel and scallop datasets (Figure 3). Low polymorphism values could explain a lower variation in the number of loci in the green crab catalogs when varying *M* and *n* parameters, as only few polymorphic loci or haplotypes would be excluded by allowing a too low number of heterozygous positions per locus (*M*) or SNPs per catalog locus (*n*) and the risk of over merging individual or catalog loci at the tested combinations would be low. Scallop and mackerel datasets instead, show higher levels of polymorphism and variation in the number of SNPs per locus between the two different tested combinations of *M* and *n*. In these cases, testing different parameter combinations could become of major importance.

**FIGURE 4:**
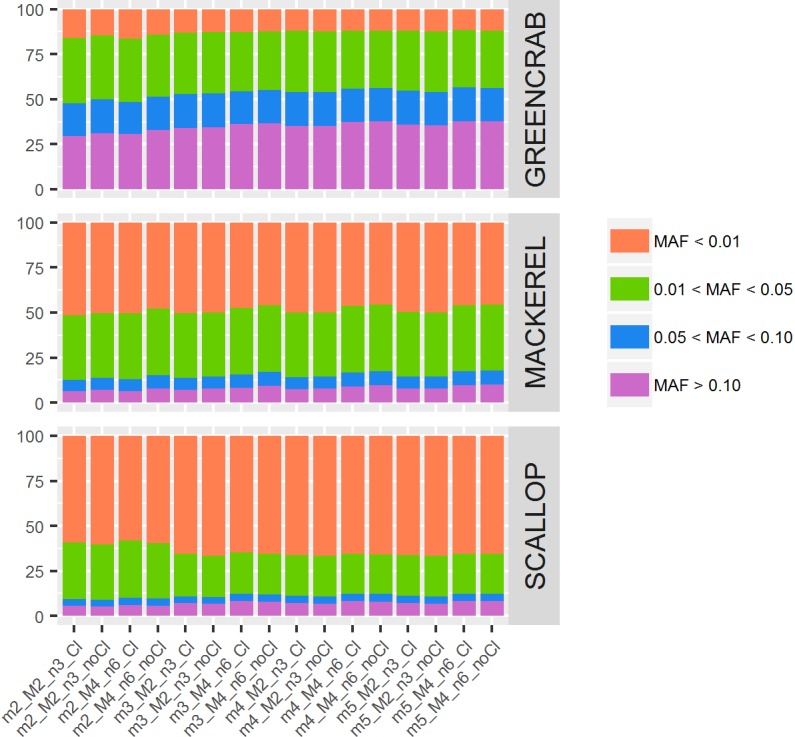
Percentage of SNPs for each MAF value range. Colored bars represent percentages of SNPs per MAF value range: orange bars indicate MAF below 0.01; green bars, MAF between 0.01 and 0.05; blue bars, MAF between 0.05 and 0.10; and purple bars, MAF higher than 0.10. Each column represents a different catalog, obtained with different values of *m*, *M*, and *n*, before (Cl) and after (noCl) filtering PCR clones.

Between datasets, proportions of SNPs with MAF values ranging between 0–0.01, 0.01–0.05, 0.05–0.10, and >0.10 vary: proportions of SNPs with MAF values below 0.01 are <17% in the green crab dataset catalogs, 45–51% in the mackerel catalogs and 58–67% in the scallop catalogs. Between catalogs within the same dataset, although proportions of SNPs relying within these MAF range categories are very similar, some differences can be observed (Figure 4). In general, with higher values of *m* and *M*/*n*, numbers of SNPs with MAF higher than 0.10 increase, while those with MAF lower than 0.01 decrease. The exception is the scallop dataset where proportion of SNPs with MAF lower than 0.01 increase in catalogs with higher values of *m* (Figure 4). The filtering of PCR clones, particularly with low values of *m*, also provided with proportionally slightly more SNPs with MAF > 0.10 in green crab and mackerel datasets. The presence of clonal reads may lead into PCR errors considered as true alleles (Andrews et al., 2016), which would not be shared among individuals, and therefore would show very low allele frequencies. Besides, their presence would be enhanced when setting low values of *m*. MAF proportions could vary due to the dataset individual compositions and their genetic distances, because of what De la Cruz and Raska (2014) call “scale” effect: rare variants would be shared at an smaller scale. They concluded that looking at structure inferred from rarer variants (lower MAF values) will show differences at a smaller scale, shared by closer located individuals, while common variants (higher MAF values) will be shared by individuals from longer distances. Therefore, the exploration of population structure at different MAF values could be informative.

### RAD-Loci Assembly and SNP Selection Parameters Affect Population Differentiation Inferences

**FIGURE 5:**
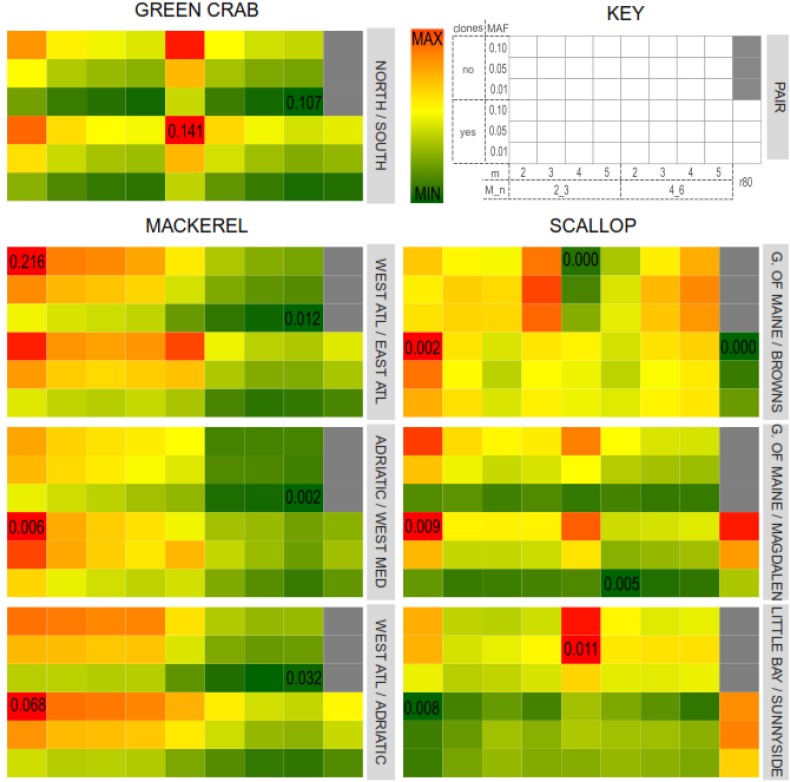
Average pairwise *F*_ST_ values for each catalog and population pairs for the three datasets: Northern/Southern green crab locations; Western/Eastern Atlantic Ocean, Adriatic Sea/Western Mediterranean Sea (intra-Mediterranean) and Western Atlantic/Adriatic Sea mackerel populations; Gulf of Maine/Magdalen Islands (northern/southern locations), Little Bay/Sunnyside (intra-North) and Gulf of Maine/Browns Bank (intra-South) scallop locations. Color gradients represent *F*_ST_ values, from lowest (dark green) to highest (dark red). Minimum and maximum *F*_ST_ values for each pair are indicated in the corresponding cell.

For all the green crab and mackerel population pairs and for the north vs. south scallop populations pairs, highest *F*_ST_ values were obtained when *m* = 2. In general, *F*_ST_ values decreased with higher values of *m* (Figure 5). This also agrees with Mastretta-Yanes et al. (2015) where catalogs with lower values of *m* resulted in higher *F*_ST_ values. Variation in *M/n* combinations had a noticeable effect in the mackerel dataset, where setting *M* = 2, *n* = 3 provided with higher *F*_ST_ values, while having little effect in the other two datasets. Besides, in the scallop intra-south and intra-north population pairs, variation of *m*, *M* and *n* do not show a clear pattern in the effect on *F*_ST_ values.

The presence of PCR clones also affected differently each dataset and population pair. Catalogs where PCR clones were kept provided with higher (in the green crab dataset and in the mackerel dataset for the Adriatic Sea/Western Mediterranean Sea and Adriatic Sea/Western Atlantic population pairs), lower (in the scallop dataset Gulf of Main/Magdalen Island and Little Bay/Sunnyside populations pairs), or more heterogeneous (in the mackerel dataset Bay of Biscay/East Canada and in the Scallop Gulf of Main/ Browns Bank population pairs) *F*_ST_ values compared to their clone-filtered relatives (Figure 5).

For each dataset, those parameters that resulted in a higher variation in the number of shared polymorphic loci, are also those with a higher effect on the estimated *F*_ST_ values. Thus, major differences were found among green crab catalogs when varying *m*, and among mackerel and scallop catalogs when varying *M* and *n*. Nevertheless, while the inferred *F*_ST_ values varied affected by the different combination of Stacks parameters tested in this study or by the filtering of PCR clones, this variation does not follow the same patterns as the number of shared polymorphic loci, nor as the number of SNPs.

Besides, the *F*_ST_ values estimated from the SNP sets from the “optimum catalogs” obtained following the “r80 rule” (Paris et al., 2017), were not the highest if compared with the rest of the catalogs which include PCR clones, except for the scallop north vs. south and intra-south population pairs (Figure 5). Mastretta-Yanes et al. (2015) found that highest mean pairwise *F*_ST_ values were obtained from the catalogs with the smallest SNP error rate (estimated by comparing sample replicates) and larger number of loci. In our datasets, we did not find any correlation between *F*_ST_ values and number of loci, which means that if minimum SNP error rates were associated with highest *F*_ST_ values, they would not be necessarily always associated with larger numbers of loci. Higher filtering thresholds for MAF values provide with larger *F*_ST_ values for the across Atlantic mackerel and scallop and green crab north vs. south population pairs (population pairs with previous evidence of genetic differentiation). Hendricks et al. (2018) also found a general trend toward increasing *F*_ST_ values with increasing MAF filtering thresholds. However, for intra-south or intra-north scallop pairs and the intra-Mediterranean Sea mackerel populations pairs it is not always the case (Figure 5). In these latter pairs, MAF values have less effect on *F*_ST_ value variation than other parameters, whereas in the former pairs, the MAF filtering threshold is the main factor affecting *F*_ST_. This agrees with De la Cruz and Raska (2014), who obtained different *F*_ST_ values when using different MAF filtering thresholds over the same SNP set. They concluded that using higher MAF thresholds (common variants) more distantly shared variants would be addressed, and therefore population structural signal could be better observed. However, for those more recently coalesced population pairs, genetic differentiation would be more likely represented by rarer variants with lower MAF values. In order to test if the obtained *F*_ST_ values were affected by the number of filtered SNPs, *F*_ST_ values estimated using subsets of 2,000 SNPs from each dataset and were found to vary following the same pattern (Supplementary Figure S6).

## Conclusion

Here we show that inferences of population differentiation based on RAD-seq derived SNPs are affected by the presence of PCR clones, RAD-loci assembly parameters and MAF threshold used for SNP selection. Importantly, different species, geographic scales and group pairs are differently affected by these factors, suggesting that the use of a systematic method based on common criteria for parameter selection might lead to limited information about genetic differentiation. Here, we show that the systematic protocol developed by Paris et al. (2017) to maximize the number of shared polymorphic loci does not necessarily imply maximizing the number of population differentiation informative markers. Yet, neither higher number of shared loci between individual, nor higher *F*_ST_ values or estimated genetic distances between *a priori* differentiated populations indicate a more realistic assemblage of RAD-seq data. For that reason, the most appropriate set of loci assembly parameters will depend on the aim of the study and different combinations should be checked for consistency (Díaz-Arce et al., 2016; Rodríguez-Ezpeleta et al., 2016) and/or be based on particular characteristics of each dataset (Rochette and Catchen, 2017). Our results suggest that those Stacks assembly parameters with highest effect on numbers of recovered shared polymorphic loci and SNPs also provide with highest variation in inferred population differentiation values. We recommend testing for different combinations of loci assembly parameters emphasizing variation of those parameters. In our study we used the Stacks software (Catchen et al., 2013), but our recommendations can be extrapolated to the use of other pipelines, such as pyRAD (Eaton, 2014) which allow the user to modulate analogous parameters.

## Author Contributions

ND-A and NR-E designed the study, interpreted the data, and wrote the manuscript. ND-A preformed the analyses.

## Conflict of Interest Statement

The authors declare that the research was conducted in the absence of any commercial or financial relationships that could be construed as a potential conflict of interest.
