# Supplementary material for: Selecting RAD-Seq Data Analysis Parameters for Population Genetics: The More the Better?
Source: Front Genet. 2019 May 29;10:533. doi: 10.3389/fgene.2019.00533 (PMC6549478; doi:10.3389/fgene.2019.00533)
Supplement: Supplementary file 1 [file Data_Sheet_1.pdf]

Selecting RAD-seq data analysis parameters for population genetics: the more the better?

Natalia Díaz-Arce, Naiara Rodríguez-Ezpeleta

Supplementary Figures

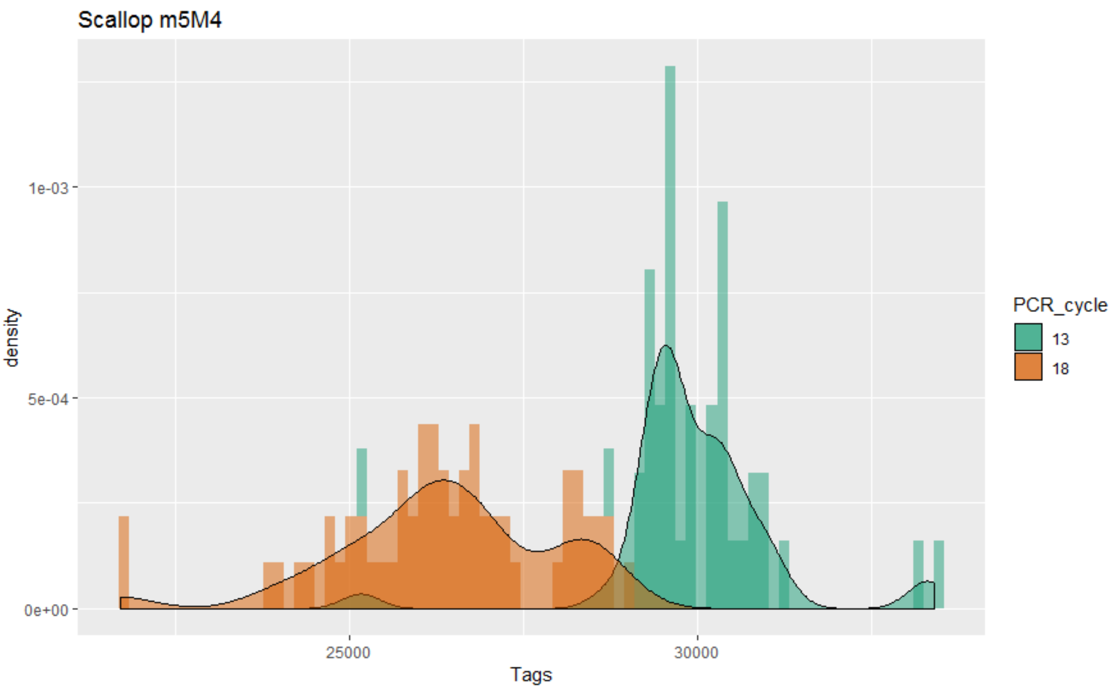

Figure S1. Frequency distribution of the number of assembled loci per individual in the Scallop dataset after applying the following parameters: *m*=5 *M*=4, and PCR clones filtered. The graph shows distribution for those samples for which 13 (green) and 18 (orange) PCR cycles were applied for library building.

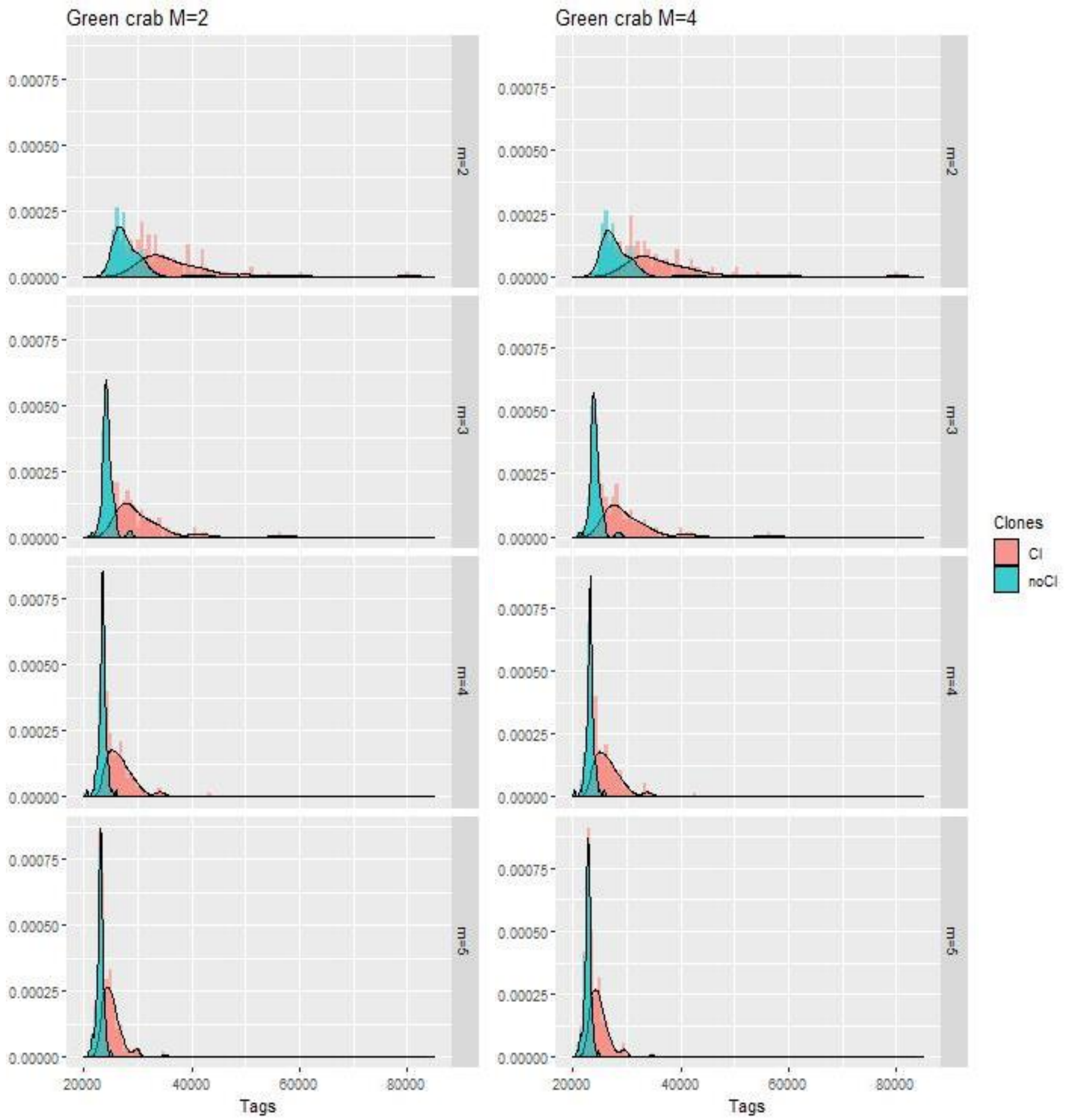

Figure S2. Frequency distribution of the number of tags per individual before (red) and after (blue) removing PCR clones for the different *m* and *M* combinations for the Green crab dataset.

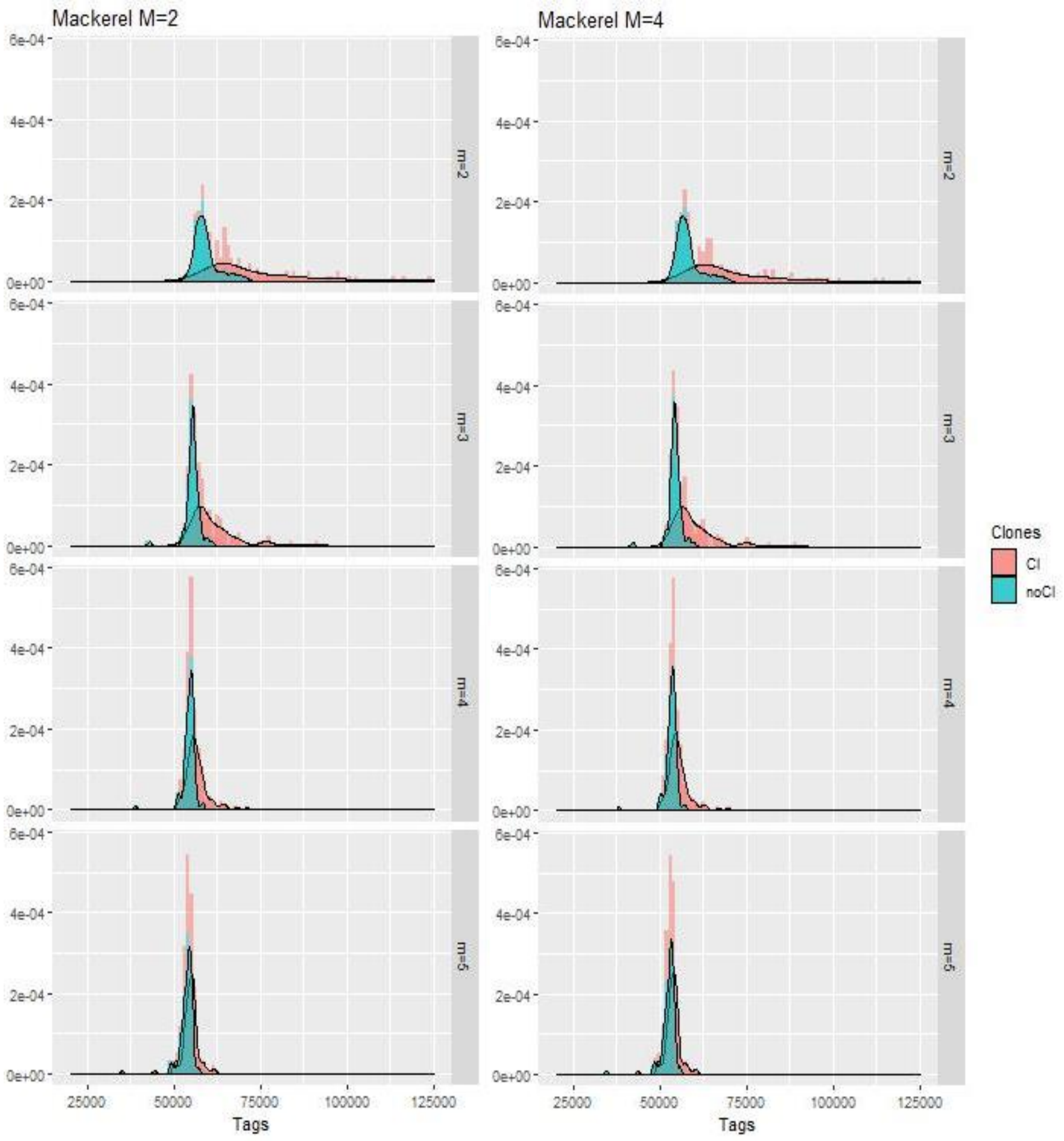

Figure S3. Frequency distribution of the number of tags per individual before (red) and after (blue) removing PCR clones for the different *m* and *M* combinations for the mackerel dataset.

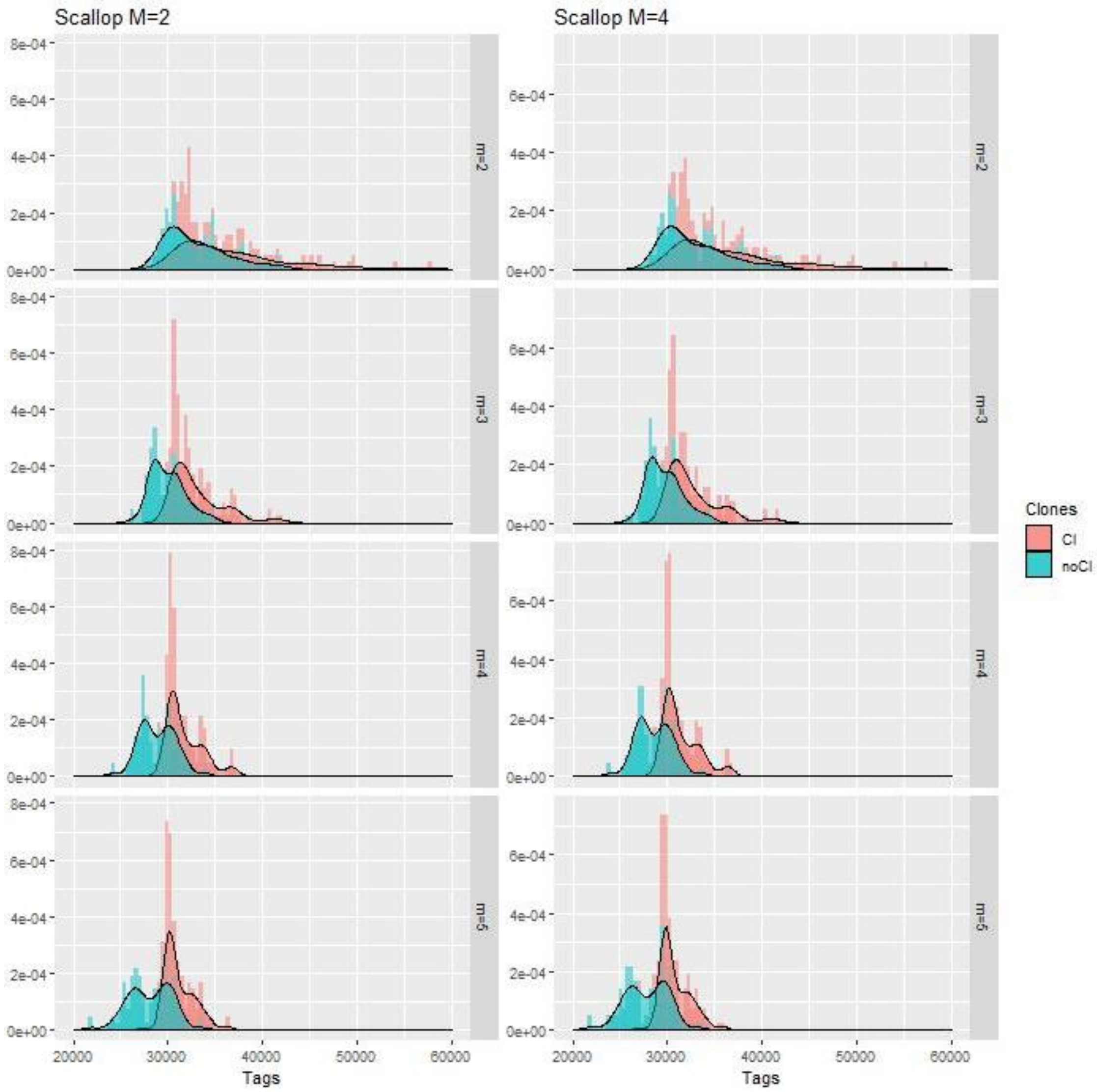

Figure S4. Frequency distribution of the number of tags per individual before (red) and after (blue) removing PCR clones for the different *m* and *M* combinations for the scallop dataset.

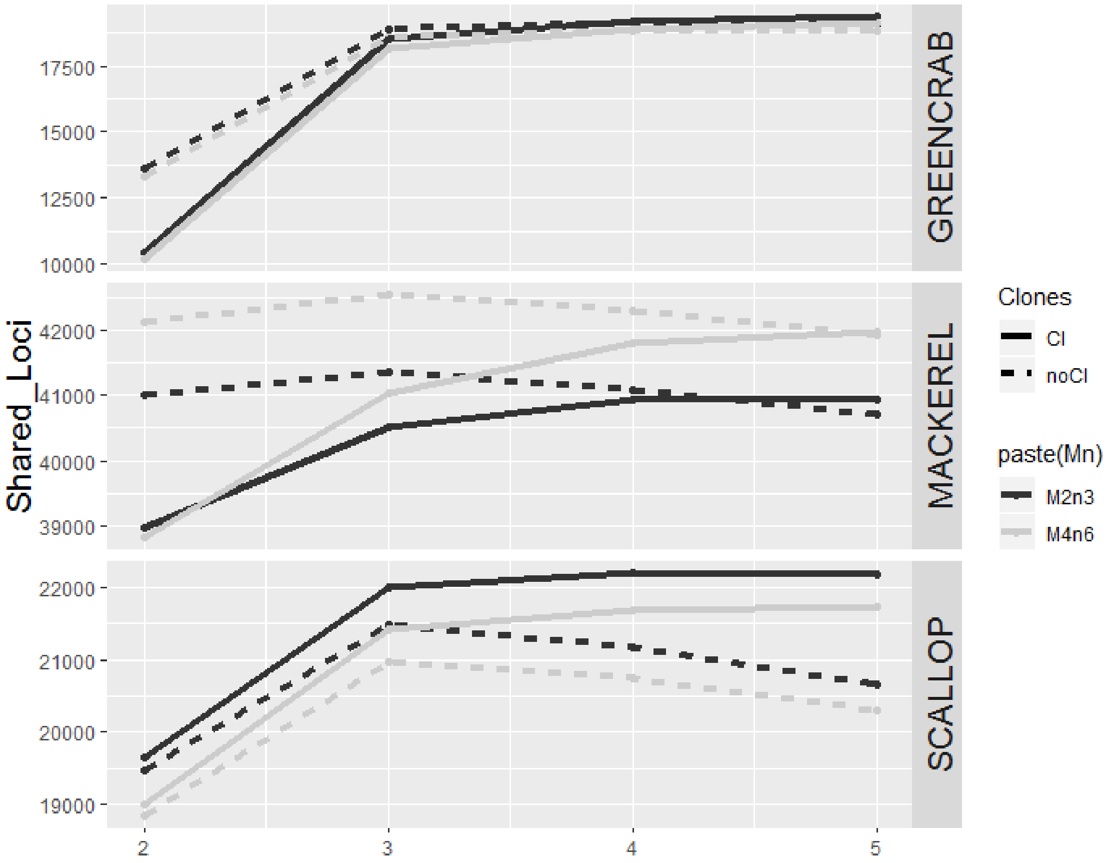

Figure S5. Total number of loci (both polymorphic and monomorphic) present in at least 75% of the individuals in all catalogs for different values of m (x axis), different combinations of M and n parameters (M=2 n=3 in black and M=4 n=6 in grey) and before (solid line) and after (dotted line) removing PCR clones.

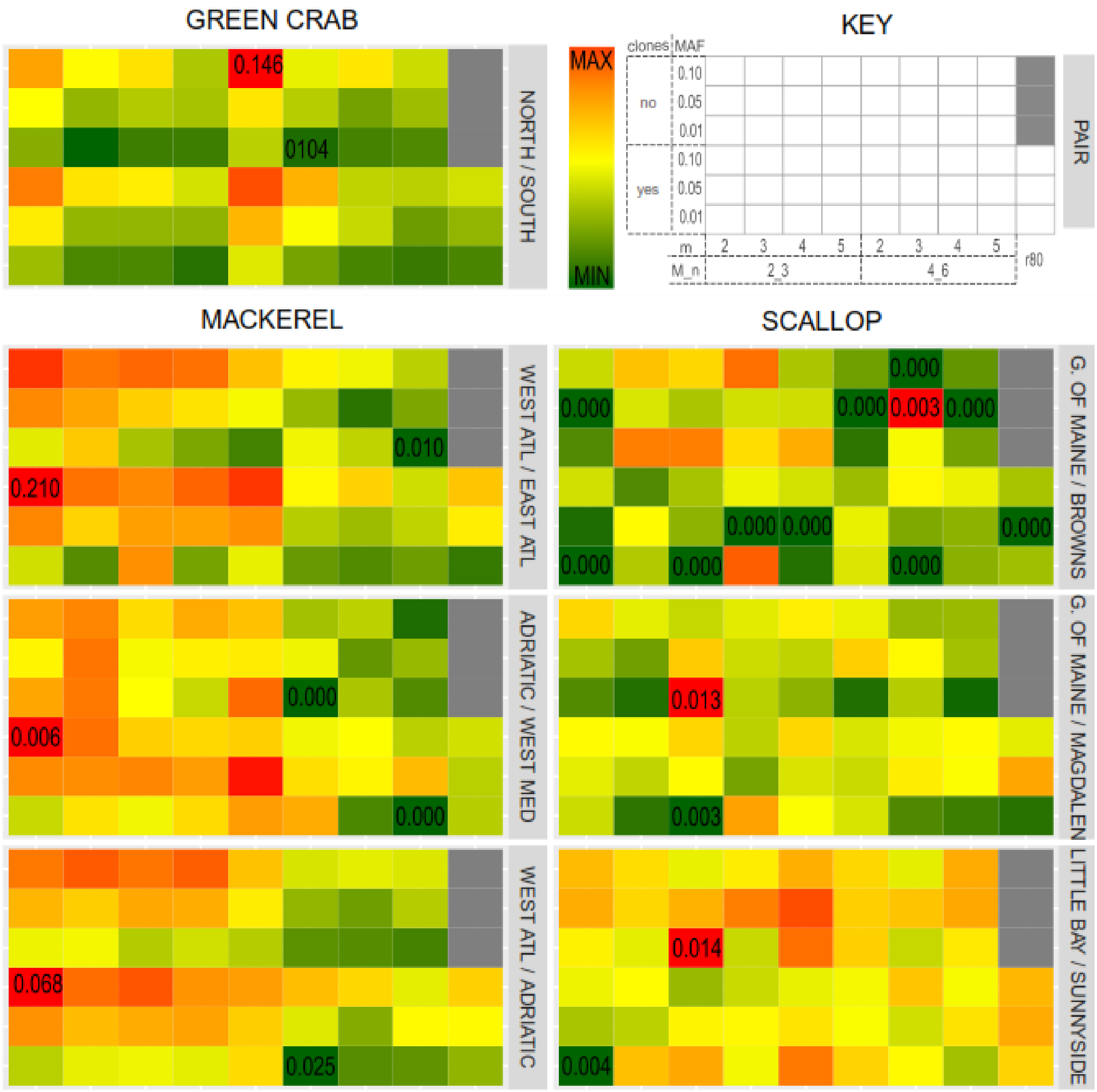

Figure S6. Average pairwise F_{ST} values for 2000 randomly selected SNPs from each catalog and population pairs for the three datasets: Northern/Southern green crab locations; Western/Eastern Atlantic Ocean, Adriatic Sea/Western Mediterranean Sea (intra-Mediterranean) and Western Atlantic/Adriatic Sea mackerel populations; Gulf of Maine/Magdalen Islands (northern/southern locations), Little Bay/Sunnyside (intra-North) and Gulf of Maine/ Browns Bank (intra-South) scallop locations. Color gradients represent F_{ST} values, from lowest (dark green) to highest (dark red). Minimum and maximum F_{ST} values for each pair are indicated in the corresponding cell.

.
